# Associations between intimate partner violence profiles and mental health among low-income, urban pregnant adolescents

**DOI:** 10.1186/s12884-019-2256-0

**Published:** 2019-04-26

**Authors:** Jordan L. Thomas, Jessica B. Lewis, Isabel Martinez, Shayna D. Cunningham, Moiuri Siddique, Jonathan N. Tobin, Jeannette R. Ickovics

**Affiliations:** 1 Department of Psychology, University of California, Los Angeles (UCLA), Los Angeles, CA USA; 2 Yale School of Public Health, New Haven, CT USA; 3 Clinical Directors Network (CDN), New York, NY USA; 4 The Rockefeller University Center for Clinical and Translational Science, New York, NY USA

**Keywords:** Intimate partner violence, Pregnancy, Adolescents, Bilateral violence, Depression, Mental health

## Abstract

**Background:**

Intimate partner violence (IPV) during pregnancy is associated with adverse maternal and child health outcomes, including poor mental health. Previous IPV research has largely focused on women’s victimization experiences; however, evidence suggests young women may be more likely to engage in bilateral violence (report both victimization and perpetration) or perpetrate IPV (unilateral perpetration) during pregnancy than to report being victimized (unilateral victimization). This study examined prevalence of unilateral victimization, unilateral perpetration, and bilateral violence, and the association between these IPV profiles and mental health outcomes during pregnancy among young, low-income adolescents.

**Methods:**

Survey data were collected from 930 adolescents (14–21 years; 95.4% Black and Latina) from fourteen Community Health Centers and hospitals in New York City during second and third trimester of pregnancy. Multivariable regression models tested the association between IPV profiles and prenatal depression, anxiety, and distress, adjusting for known predictors of psychological morbidity.

**Results:**

Thirty-eight percent of adolescents experienced IPV during their third trimester of pregnancy. Of these, 13% were solely victims, 35% were solely perpetrators, and 52% were engaged in bilateral violence. All women with violent IPV profiles had significantly higher odds of having depression and anxiety compared to individuals reporting no IPV. Adolescents experiencing bilateral violence had nearly 4-fold higher odds of depression (OR = 3.52, 95% CI: 2.43, 5.09) and a nearly 5-fold increased likelihood of anxiety (OR = 4.98, 95% CI: 3.29, 7.55). Unilateral victims and unilateral perpetrators were also at risk for adverse mental health outcomes, with risk of depression and anxiety two- to three-fold higher, compared to pregnant adolescents who report no IPV. Prenatal distress was higher among adolescents who experienced bilateral violence (OR = 2.84, 95% CI: 1.94, 4.16) and those who were unilateral victims (OR = 2.21, 95% CI: 1.19, 4.12).

**Conclusions:**

All violent IPV profiles were associated with adverse mental health outcomes among pregnant adolescents, with bilateral violence having the most detrimental associations. Comprehensive IPV screening for both victimization and perpetration experiences during pregnancy is warranted. Clinical and community prevention efforts should target pregnant adolescents and their partners to reduce their vulnerability to violence and its adverse consequences.

**Trial registration:**

ClinicalTrials.gov, NCT00628771. Registered 29 February 2008.

## Introduction

Intimate partner violence (IPV) is a pervasive public health problem, impacting an estimated 4.7 million women and 5.4 million men annually [1]. Adolescent women are at particularly high risk, with nearly 70% of IPV victims experiencing their first IPV incident before age 25, and nearly one quarter before age 18 [1]. Pregnant adolescents have significantly higher rates of IPV than older mothers and their non-pregnant peers [2–7]. Pregnant adolescents are engaged simultaneously in the developmental tasks of adolescence and the transition to parenthood—both of which are stressful, and likely increase their vulnerability [8–10].

Among both adolescents and adult women, IPV during pregnancy is associated with adverse maternal and child health outcomes including psychological morbidity [5, 6, 11–16], pregnancy complications (e.g., vaginal bleeding, kidney infection, urinary tract infection, low gestational weight gain, preterm birth, low birth weight) [17–23], and risky perinatal health behaviors [22–30]. Research has shown that low-income pregnant and parenting adolescents are particularly vulnerable to the burden of postpartum depressive symptoms [7]. Although findings have been inconsistent [31], some studies suggest there are higher rates of perinatal depression and other adverse mental health outcomes among racial/ethnic minority women [32, 33]. In addition, Black and Latina women have a higher twelve-month prevalence of rape, physical violence or stalking by an intimate partner than do white women [34], indicating this population may be particularly vulnerable to IPV and its related health outcomes [35].

Historically, IPV studies have focused largely on women’s victimization experiences and outcomes [36]. However, evidence suggest that more than one-half of reported IPV could be categorized as bilateral, involving victimization and perpetration by both partners [37]. As a result, more recent research has begun to examine distinct profiles of intimate partner violence (i.e., unilateral victimization, unilateral perpetration, bilateral violence) and their correlates [38–42]. The limited studies of pregnant adolescents consistently find that more young women report perpetrating IPV during pregnancy than report being victimized [18, 40, 41, 43–45]. Perpetration of IPV has been linked to adverse mental health outcomes for women in previous studies [18, 38, 43, 45]. Moreover, those in bilaterally violent relationships may be at highest risk for adverse maternal and child outcomes [18, 40]. More research on differing IPV profiles is needed in diverse populations and settings to inform screening and intervention strategies.

The objectives of this study are to (1) assess the prevalence of unilateral victimization, unilateral perpetration, and bilateral violence; and (2) determine whether these IPV profiles are differentially associated with depression, anxiety, and prenatal distress during pregnancy among young, low-income adolescents. We hypothesized that bilateral violence would be associated with the greatest risk of adverse mental health consequences.

## Methods

### Participants

Data for this study were obtained from a cluster randomized trial of group prenatal care designed to improve maternal-child health and reduce HIV risk behaviors [46]. Pregnant adolescents aged 14 to 21 years (*N* = 1233) were recruited from 14 Community Health Centers and hospital obstetric outpatient settings in New York City between 2008 and 2011, with follow-up data collection completed in 2012. To participate, women had to be less than 24 weeks gestation at study entry, not at high-risk obstetrically, and fluent in either English or Spanish. The cohort for this secondary data analysis is limited to adolescents with complete data on IPV during the third trimester of pregnancy, resulting in an analytic sample of *N* = 930 participants. Those excluded from this secondary data analysis were slightly older (M = 18.84 years, SD = 1.66) than those included (M = 18.62 years, SD = 1.75) and were less likely to have been born outside the United States. There were no other significant sociodemographic differences.

### Procedures

Participants completed structured interviews via audio-handheld assisted personal interview technology at four points across the perinatal period, including the second and third trimester and six and twelve months postpartum. Interviews were approximately forty minutes and were completed in either English or Spanish. Analyses for this paper use data collected during the second (14–24 weeks gestation) and third (32–42 weeks gestation) trimesters of pregnancy. Participants were paid $20 for each interview. All procedures were approved by Institutional Review Boards (IRBs) at Yale University, Clinical Directors Network, and each clinical site.

### Primary predictor variable

#### Intimate partner violence

IPV profiles for participants were created, using data from an adapted form of the Revised Conflict Tactics Scale [47] collected during their third trimester of pregnancy. Participants were asked to self-report the frequency of both victimization and perpetration experiences related to psychological (e.g., insult, swear, threaten), physical (e.g., push, grab, shove, slap, kick, bite punch, beat up, burn, choke), and sexual (e.g., forced sex, forced sexual acts) violence in their romantic relationships since their baseline (second trimester) interview. Individual item responses were coded dichotomously (yes/no) to identify any experience of IPV for each item. Separate variables were then created for each type of IPV (e.g., psychological, physical, sexual). Following previous research [30, 48, 49], responses were then categorized by experiences of any IPV victimization and any IPV perpetration. Using these dichotomous variables of victimization and perpetration, a new variable was created to represent four “intimate partner violence [IPV] profiles”: (1) participants who reported no IPV victimization and no IPV perpetration (no IPV); (2) participants who reported IPV victimization but no IPV perpetration (unilateral victimization); (3) participants who perpetrated IPV and who reported no IPV victimization (unilateral perpetration); and (4) participants who reported both IPV victimization and IPV perpetration (bilateral violence).

### Dependent variables

#### Depression

Depression was measured using the Center for Epidemiologic Studies Depression Scale (CES-D) [50]. As in prior studies of pregnant women [31], five somatic items were excluded from the 20-item scale to avoid overlap with pregnancy symptoms (e.g., changes in appetite or sleep). This affect-only adaptation includes 15 items regarding frequency of depressed mood (e.g., “feel depressed,” “feel lonely”) in the past week. Responses include less than one day (0), one to two days (1), three to four days (2) and five to seven days (3). Responses were reverse coded as needed, and summed to create a composite score for depression where greater scores indicated higher levels of depressive symptoms (range 0–45). A cutoff of greater than or equal to 16 is typically used to classify cases of depression for the full CES-D (range 0–60). For this study, participants with a CES-D score greater than or equal to 12 were classified as clinically depressed to account for truncated version of the scale (i.e., ratio based on maximum possible total score). Similar approaches have been used among other studies of young pregnant and postpartum couples [51, 52].

#### Anxiety

Anxiety was measured using the Generalized Anxiety Disorder Scale (GAD-7) [53]. The GAD-7 includes seven items for self-reporting frequency of anxiety-related problems in the previous two weeks. Items are summed and result in a composite score ranging from 0 to 21, with higher scores indicating higher levels of anxiety-related problems. As per clinical guidelines, a score of greater than or equal to 10 was used to identify cases of moderate anxiety [53].

#### Prenatal distress

Prenatal distress was measured using the Revised Prenatal Distress Questionnaire (PDQ) [54, 55]. Participants were asked “how much are you bothered, worried, or upset” about 17 issues associated specifically with pregnancy (e.g., low energy, physical symptoms such as nausea or backaches, changes in weight/body shape, and taking care of the newborn baby) on a three-point scale ranging from not at all (0) to very much (2). Responses were summed to create a total prenatal-specific distress score, and following previous research [56], dichotomized based on the median split (11) to represent high and low prenatal distress in the sample. Other literature has reported similar mean values (9) among comparable study samples [57].

#### Covariates

Data obtained from participants included the following covariates known to be associated with depression, anxiety, or pregnancy distress: age, education (some high school, high school graduate, some college), employment status (employed, unemployed), relationships status (married or living together versus single/never married, separated/divorced, widowed), nativity (US versus foreign born), race/ethnicity (Hispanic/Latina, non-Latina Black, Other), and parity (nulliparous versus not).

### Analyses

Descriptive statistics (means, frequencies) were calculated using ANOVA and chi-square tests to determine if women with different IPV profiles differed significantly on sociodemographic or psychosocial characteristics. Bivariate and multivariable logistic regression models were conducted to determine likelihood of depression, anxiety, and prenatal distress for each IPV profile with “no IPV” as the reference category. All covariates were evaluated as potential confounders in multivariable models. Variables with significance of α = 0.05 or lower were kept in multivariable models. Analyses also controlled for study condition (i.e., assignment to group versus individual care). Analyses were completed using SAS 9.4 and IBM SPSS 25 software.

## Results

### Sample characteristics

Table 1 contains the distribution of sociodemographic and psychosocial characteristics in this sample by IPV profile. Overall, 38.8% (*n* = 361) of the sample reported experiencing some form of IPV during the prenatal period. Of these, 13% were only victims, 35% were only perpetrators and 52% were engaged in bilateral violence as both victim and perpetrator. These relationships were further characterized by the type of violence (e.g., psychological, physical, and sexual) of that occurred. Bilaterally violent relationships had the highest rates of violence, regardless of type of violent act. Psychological violence was the most common across IPV profiles; 35% of all violent relationships involved verbal acts of violence. Nearly one-quarter of all violent relationships involved physical acts of violence. Overall, sexual violence was uncommon, though it occurred most frequently in bilaterally violent relationships.

**Table 1:** Sample characteristics, % (n) or M (SD)

	No Violence 61.2 (569)	Unilateral Victimization 5.2 (48)	Unilateral Perpetration 13.5 (126)	Bilateral Violence 20.1 (187)	Overall (*N* = 930)	*p*-value
Age						
Race/Ethnicity						0.001
Hispanic/Latina	66.3 (377)	50.0 (24)	62.7 (79)	48.7 (91)	61.4 (571)	
Black, non-Latina	29.5 (168)	41.7 (20)	31.7 (40)	45.5 (85)	33.7 (313)	
Other^a^	4.2 (24)	8.3 (4)	5.6 (7)	5.9 (11)	4.9 (46)	
Nativity						0.023
U.S. born	59.9 (341)	62.5 (30)	65.9 (83)	72.2 (135)	63.3 (589)	
Foreign born	40.1 (228)	37.5 (18)	34.1 (43)	27.8 (52)	36.7 (341)	
Education						0.519
Some high school	61.4 (344)	60.4 (29)	64.3 (81)	53.5 (100)	60.2 (554)	
High school graduate	20.0 (112)	18.8 (9)	18.3 (23)	22.5 (42)	20.2 (186)	
Some college	18.6 (104)	20.8 (10)	17.5 (22)	24.1 (45)	19.7 (181)	
Nulliparous						0.211
No	15.9 (86)	17.0 (8)	11.5 (14)	10.4 (19)	14.2 (127)	
Yes	84.1 (455)	83.0 (39)	88.5 (108)	89.6 (164)	85.8 (766)	
Employment						0.209
Unemployed	80.0 (453)	85.4 (41)	75.2 (94)	74.7 (139)	78.6 (727)	
Employed	20.0 (113)	14.6 (7)	24.8 (31)	25.3 (47)	21.4 (198)	
Main source of financial support						0.051
Self	18.5 (105)	20.8 (10)	21.6 (27)	18.8 (35)	19.1 (177)	
Partner	32.5 (183)	29.2 (14)	21.6 (27)	25.8 (48)	29.3 (272)	
Parent	33.6 (191)	39.6 (19)	46.4 (58)	34.4 (64)	35.8 (332)	
Other	15.7 (89)	10.4 (5)	10.4 (13)	21.0 (39)	15.7 (146)	
Relationship status						0.020
Married or living together	45.2 (249)	37.8 (17)	39.2 (49)	32.4 (59)	41.4 (374)	
Other relationship (single/never married, sep/divorced, widowed)	54.8 (302)	62.2 (28)	60.8 (76)	67.6 (123)	58.6 (529)	
Depression						< 0.0001
Not depressed	67.8 (386)	45.8 (22)	53.2 (67)	39.6 (74)	59.0 (549)	
Depressed	32.2 (183)	54.2 (26)	46.8 (59)	60.4 (113)	41.0 (381)	
Anxiety						< 0.0001
No	88.4 (502)	78.7 (37)	78.6 (99)	61.0 (114)	81.0 (752)	
Yes	11.6 (66)	21.3 (10)	21.4 (27)	39.0 (73)	19.0 (176)	
Prenatal distress						< 0.0001
Low	52.5 (297)	33.3 (16)	45.6 (57)	27.0 (50)	45.5 (420)	
High	47.5 (269)	66.7 (32)	54.4 (68)	73.0 (135)	54.5 (504)	
Type of violence						< 0.0001
Physical	–	37.5 (18)	44.4 (56)	72.7 (136)	22.6 (210)	
Verbal	–	87.5 (42)	83.3 (105)	96.3 (180)	35.2 (327)	
Sexual	–	14.6 (7)	1.6 (2)	15.5 (29)	4.1 (38)	

^a^Includes non-Hispanic White, American Indian or Alaska Native, Asian, Native Hawaiian or Other Pacific Islander, and non-disclosed Other

Overall, 41% (*n* = 381) of the sample was depressed, 19% (*n* = 176) reported anxiety, and 54.5% (*n* = 504) reported high prenatal distress. Within IPV profiles, depression, anxiety, and prenatal distress were lowest among those with no IPV, followed by unilateral perpetrators, unilateral victims, and highest among those reporting bilateral violence.

### Association between IPV experiences and mental health outcomes

In bivariate analyses, all violent IPV profiles had significantly higher odds of having depression, anxiety and pregnancy distress compared to individuals reporting no IPV. Relative to individuals experiencing no IPV, the odds of having depression (OR_Bilat IPV_ = 3.22, 95% CI: 2.29, 4.53), anxiety (OR_Bilat IPV_ = 4.87, 95% CI: 3.30, 7.19), and prenatal distress (OR_Bilat IPV_ = 2.98, 95% CI: 2.08, 4.29) were highest amongst the bilateral violence group for all outcomes.

Table 2 shows the unadjusted and adjusted results of the multivariable models for associations between IPV and mental health. Compared to individuals who experienced no IPV, the odds of having depression were more than 2-fold higher for unilateral perpetrators (OR = 2.05, 95% CI: 1.36, 3.09), 3-fold higher for unilateral victims (OR = 3.03, 95% CI: 1.60, 5.73), and almost 4-fold higher for those reporting bilateral violence (OR = 3.52, 95% CI: 2.43, 5.09). All IPV profiles likewise had a significantly higher likelihood of reporting anxiety relative to individuals who experienced no IPV: a more than 2-fold increase for unilateral perpetrators (OR = 2.14, 95% CI: 1.28, 3.58) and unilateral victims (OR = 2.22, 95% CI: 1.03, 4.81), and a nearly 5-fold increase for those reporting bilateral violence (OR = 4.98, 95% CI: 3.29, 7.55). A similar trend for prenatal distress was observed for unilateral victims and individuals who experienced bilateral violence. Unilateral victims were more than twice as likely (OR = 2.62, 95% CI: 1.33, 5.14) and individuals who experienced bilateral violence were almost 3 times more likely (OR = 2.84, 95% CI: 1.94, 4.16) to report prenatal distress compared to individuals who experienced no IPV. Unilateral perpetrators were not at increased risk for prenatal distress. *P*-values for all significant associations ranged from *p* < 0.05—*p* < 0.0001.

**Table 2:** Associations between overall IPV profile and mental health outcomes^a^

	Depression		Anxiety		Prenatal distress	
	OR	AOR	OR	AOR	OR	AOR
No violence	1.00 (reference)	1.00 (reference)	1.00 (reference)	1.00 (reference)	1.00 (reference)	1.00 (reference)
Unilateral victimization	2.49** (1.38, 4.52)	3.03** (1.60, 5.73)	2.06 (0.98, 4.32)	2.22* (1.03, 4.81)	2.21* (1.19, 4.12)	2.62** (1.33, 5.14)
Unilateral perpetration	1.86** (1.26, 2.75)	2.05*** (1.36, 3.09)	2.07** (1.26, 3.41)	2.14** (1.28, 3.58)	1.32 (0.89, 1.94)	1.37 (0.91, 2.05)
Bilateral violence	3.22*** (2.29, 4.53)	3.52*** (2.43, 5.09)	4.87*** (3.30, 7.19)	4.98*** (3.29, 7.55)	2.98*** (2.08, 4.29)	2.84*** (1.94, 4.16)

^a^Adjusted odds ratios (AOR) control for age, education, employment status, relationship status, intervention arm, nativity, parity and race/ethnicity

*p < 0.05 **p < 0.005 ***p < 0.0001

## Discussion

Results of this study provide insight into the prevalence and correlates of physical, psychological and sexual IPV among pregnant adolescents. Prevalence of partner violence was high, with 38% of pregnant adolescents reporting some type of violence during pregnancy. Psychological violence was particularly common among violent relationships. Nearly 1 in 5 participants were involved in bilateral violence with their partner, placing the entire family at risk for adverse outcomes.

IPV among pregnant adolescents was associated with increased risk of adverse mental health outcomes for all violence profiles, regardless of victimization or perpetration status. Unilateral victims, perpetrators, and those who engaged in bilateral violence were all at significantly higher risk for depression and anxiety. Unilateral victims and those who engaged in bilateral violence were also at increased risk for prenatal distress; however, unilateral perpetrators were not. Prenatal distress measures concerns specific to pregnancy and baby wellness. It may be that fear of physical injury from IPV is driving increased prenatal distress for those whose IPV profiles include victimization. In this sample, nearly one-quarter of violent relationships were characterized by physical violence. Further, rates of psychological violence were higher among those whose IPV profiles included victimization relative to unilateral perpetrators. Psychological violence may include threats of bodily harm that may or may not be enacted. Thus, unilateral perpetrators do not have increased prenatal distress.

Adolescents engaging in bilateral violence during pregnancy had the greatest likelihood of reporting adverse mental health outcomes. Specifically, these pregnant adolescents were nearly four times more likely to experience depression, nearly five times more likely to experience anxiety, and almost three times as likely to experience prenatal distress relative to adolescents who did not experience IPV. Another study of more than 1000 high-risk African American pregnant women found that those in bilaterally violent relationships were at highest risk for depression [18]. Lewis and colleagues likewise recently documented pregnant adolescent couples with mutual IPV have the least healthy relationship (e.g., attachment anxiety, relationship equity) and psychological (e.g., depression, stress, hostility) characteristics [40]. The differential associations highlighted by each of these studies confirm the importance of screening adolescents for specific IPV profiles during pregnancy, since they are associated with varied risks for mental health.

Several factors may underlie these findings. Previous research has demonstrated that women engaging in violence often do so in the context of responding to partner-initiated violence [58, 59]. Such retaliatory responses may be intensified during pregnancy, a time of heightened socioemotional, physiologic, and behavioral change. Pregnant women may feel protective of their unborn baby [60], and motivated to incite or reciprocate violence in their relationships. Relationship aggression may be especially pronounced among adolescent mothers due to specific developmental and contextual factors, including physical, psychological, and cognitive changes, increased stress, and the transition to parenting – all of which are psychologically taxing [58, 61], and require increased access to resources.

Notably, these findings confirm that both being a victim or perpetrator of violence in an intimate relationship impacts young women’s mental health, and that bilateral violence engagement is especially psychologically harmful. It is well-established that poor mental health in pregnancy is associated with multiple adverse birth, neonatal, and infant outcomes [62, 63]. Mothers who experience depression or anxiety are more likely to engage in negative health-related behaviors and have difficulty managing infant distress [14]. Thus, the impact may extend far beyond the perinatal period, with adolescents who participate in bilateral violence at high risk for both adverse mental health in pregnancy and more long-term physical and mental health consequences [18].

Data examining women’s perpetration of IPV have been critiqued throughout the literature for undermining the adverse asymmetrical effects partner violence has on women [38]. Bidirectionality of IPV does not indicate that men and women perpetrate violence for the same reasons or experience the same effects [64]. Yet, given the associations between perpetration and mental health found in this study and others [18, 38, 43, 45], it is critical to women’s health to consider both perpetration and victimization experiences. Avenues for future research could include quantitative and qualitative studies that focus on adolescent couples’ motivations for perpetration and other contextual factors of IPV in pregnancy.

There were several limitations to this study. The analysis focused on discrete acts of violence and did not capture severity, frequency, or other contextual or relational factors. Due to the cross-sectional design, we cannot make causal inferences about these associations. IPV was based on retrospective self-report by women only and thus is subject to social desirability bias and underreporting. Future work could include partner health outcomes or couple-level health indicators, as well. Our sample was largely comprised of pregnant Black and Latina adolescents and included only a small number of non-minority women, thereby limiting the generalizability of results to women of other races and ages. Future research could examine if associations between partner violence and mental health differ by race using larger and more representative study samples.

In contrast, important study strengths include clinically-relevant effects highlighting the impact of partner violence on young women’s mental health. Additionally, few studies ask women about their own perpetration of violence. This study extends the evidence base for the relative impact of different IPV profiles on mental health. Future work should continue to build upon the terms used to describe these profiles (e.g., unilateral victimization, unilateral perpetration, and bilateral violence), as heterogeneity in IPV terminology across the literature may mask potential differences between studies and result in underestimations of effects.

The health care system represents a clear target for preventive intervention, as pregnant women have regular engagement with health care professionals throughout the perinatal period. This study provides further evidence of the need for comprehensive IPV screening as a national standard in prenatal care. Routine universal screening for IPV among women patients has been recommended by the American Congress of Obstetricians and Gynecologists and others [65–67], but existing screening tools do not comprehensively assess IPV. Emphasis on an exclusive “male perpetrator—female victim” paradigm in research and clinical treatment and prevention may be ineffective at reducing violence for pregnant adolescents. In addition to incorporating perpetration assessments into clinical screening tools, it may also be beneficial to incorporate screening and prevention efforts into other clinical and community interventions with adolescent couples and mothers (e.g., labor preparation, expecting parent skill development programs) [43].

## Conclusion

Intimate partner violence is prevalent among women of reproductive age and has the potential to contribute to adverse mental health outcomes during pregnancy and beyond. Pregnant adolescents may experience distinct IPV profiles (e.g., unilateral IPV victimization, unilateral IPV perpetration, and bilateral violence), all of which are associated with adverse mental health. Bilateral violence, the most prevalent type of IPV, appears to be associated with greater depression, anxiety, and prenatal distress than unilateral forms of violence. Future research should assess risk factors that are associated with distinct profiles and types of IPV and investigate optimal intervention strategies to reduce both victimization and perpetration during pregnancy, a period during which couples may be motivated to engage in behavior change on behalf of their future family. A comprehensive understanding of the associations between IPV profiles and mental health is needed to tailor prevention and intervention strategies for young pregnant and parenting couples, mitigating health risks related to partner violence among this vulnerable population.

## Abbreviations

ANOVA: Analysis of variance
AOR: Adjusted odds ratio
CES-D: Center for Epidemiologic Studies Depression
CI: Confidence interval
GAD: Generalized anxiety disorder
HIV: Human immunodeficiency virus
IPV: Intimate partner violence
IRB: Institutional Review Board
OR: Odds ratio
PDQ: Prenatal Distress Questionnaire
